# Novel Bacteriophages Capable of Disrupting Biofilms From Clinical Strains of *Aeromonas hydrophila*

**DOI:** 10.3389/fmicb.2020.00194

**Published:** 2020-02-14

**Authors:** Mwila Kabwe, Teagan Brown, Lachlan Speirs, Heng Ku, Michael Leach, Hiu Tat Chan, Steve Petrovski, Peter Lock, Joseph Tucci

**Affiliations:** ^1^Department of Pharmacy and Biomedical Science, La Trobe Institute for Molecular Science, La Trobe University, Melbourne, VIC, Australia; ^2^School of Rural Health, Monash University, Bendigo, VIC, Australia; ^3^Department of Microbiology, The Royal Melbourne Hospital, Melbourne, VIC, Australia; ^4^Department of Physiology, Anatomy and Microbiology, La Trobe University, Melbourne, VIC, Australia; ^5^La Trobe Institute for Molecular Science, La Trobe University, Melbourne, VIC, Australia

**Keywords:** *Aeromonas hydrophila*, bacteriophage, genomics, biofilm, antimicrobial resistance

## Abstract

The increase in global warming has favored growth of a range of opportunistic environmental bacteria and allowed some of these to become more pathogenic to humans. *Aeromonas hydrophila* is one such organism. Surviving in moist conditions in temperate climates, these bacteria have been associated with a range of diseases in humans, and in systemic infections can cause mortality in up to 46% of cases. Their capacity to form biofilms, carry antibiotic resistance mechanisms, and survive disinfection, has meant that they are not easily treated with traditional methods. Bacteriophage offer a possible alternative approach for controlling their growth. This study is the first to report the isolation and characterization of bacteriophages lytic against clinical strains of *A. hydrophila* which carry intrinsic antibiotic resistance genes. Functionally, these novel bacteriophages were shown to be capable of disrupting biofilms caused by clinical isolates of *A. hydrophila.* The potential exists for these to be tested in clinical and environmental settings.

## Introduction

*Aeromonas hydrophila* is a Gram-negative rod found in fresh water, brackish water, and mud in temperate climates (Batra et al., 2016). They are an established fish pathogen causing septicemia and ulcerative diseases (Chowdhury et al., 1990). *Aeromonas* spp. were first reported as infective agents in humans in 1951, and from that time have been seen as important human pathogens (Janda and Abbott, 2010). *A. hydrophila* implicated in human infections is usually mesophilic and grows optimally between 35 and 37°C (Janda and Abbott, 2010). Clinical infections may be a result of direct skin invasion from muddy water (Vally et al., 2004) or drinking contaminated water leading to local (gastritis, skin necrotizing infection) or systemic (peritonitis, sepsis, meningitis, respiratory, and hepatic) infections (Janda and Abbott, 2010; Igbinosa et al., 2012). Mortality in systemic infections may be as high as 46% (Dryden and Munro, 1989).

The incidence of *A. hydrophila* has been correlated to warmer summer periods when the prevalence of this bacterium is increased in harvested rain water (Picard and Goullet, 1987) and in chlorinated or unchlorinated metropolitan water supplies (Burke et al., 1984a, b).

Global climate change and population increases are expected to put greater pressures on water resources (DeNicola et al., 2015) and lead to increased investment in alternative sources such as rain harvesting (Ahmed et al., 2008). This is certainly the case in countries such as Australia, that is experiencing harsher summers and where use of recycled water for human consumption is not in vogue. *A. hydrophila* is estimated to be present in up to 33% of rain harvested water in Australia’s major cities (Chubaka et al., 2018) and they are known to survive in chlorinated water by forming biofilms (Igbinosa et al., 2012). Biofilms provide bacterial cell-to-cell contact allowing for the transfer of genetic material that enhances the niche and increases resistance to stress and antibiotics (Talagrand-Reboul et al., 2017). Therefore, water originating from rain harvested tanks, municipal supplies, recreational settings such as swimming pools, and in the natural environment may serve as potential sources of infection.

*Aeromonas* spp. play a major role in the transfer of antibiotic resistance, making these organisms particularly problematic. They have been implicated as mediators of the transfer of antibiotic resistance markers between hospital and environmental strains (Varela et al., 2016), and are associated with innate multi-antibiotic resistance due to efflux pumps, inducible cephalosporinases, and inducible metallo beta lactamases (Azzopardi et al., 2011; Sinclair et al., 2016). Controlling *A. hydrophila* infection is therefore paramount as this organism threatens food security (by causing fish diseases and increasing their mortality) (Talagrand-Reboul et al., 2017) and human health (Neyts et al., 2000). Since the World Health Organization declaration that antibiotic resistance was a global emergency (World Health Organisation [WHO], 2014), alternatives for antibiotics have been actively researched (Czaplewski et al., 2016).

Bacteriophages, although discovered before antibiotics, have recently emerged as adjuncts and alternatives to antibiotics (Golkar et al., 2014). While temperate (Beilstein and Dreiseikelmann, 2008; Dziewit and Radlinska, 2016) and lytic (Chow and Rouf, 1983; Merino et al., 1990a, b; Shen et al., 2012; Jun et al., 2013; Anand et al., 2016; Wang et al., 2016; Le et al., 2018; Yuan et al., 2018) bacteriophages against *A. hydrophila* have been previously reported, these were isolated using environmental and fish pathogenic isolates of *A. hydrophila*. In the instances where host range was tested, their activity did not extend to clinical strains of *A. hydrophila* (Wang et al., 2016), that is, strains which were isolated from hospital patients suffering from *A. hydrophila* infections. Clinical strains of *A. hydrophila* have been shown to differ from environmental strains including those pathogenic in fish, in their production of virulence factors (Janda and Abbott, 2010), as well as other features. For instance, clinical strains are reported to restrict production of protease activity in favor of cytotoxicity and hemolysin production when temperatures increase from 30 to 37°C (Yu et al., 2007; Rasmussen-Ivey et al., 2016). Further, environmental strains can survive at temperatures as low as 4°C where clinical strain growth is inhibited (Mateos et al., 1993; Rasmussen-Ivey et al., 2016). To date there have been no lytic bacteriophages isolated that have demonstrated killing of clinical strains of *A. hydrophila*. This study screened for lytic bacteriophages against clinical strains of *A. hydrophila* associated with various human diseases. The isolated bacteriophages were characterized phenotypically and genomically, and functionally assessed for their capacity to degrade *A. hydrophila* biofilms.

## Materials and Methods

### Ethics Statement

All methods were performed in accordance with the La Trobe University Ethics, Biosafety, and Integrity guidelines and regulations. Clinical isolates of *A. hydrophila* were obtained from specimen cultures as part of routine care. Informed consent was obtained from participants for their involvement and use of samples in this study. The study protocols were approved by the La Trobe University Ethics Committee, reference number: S17–111.

### Bacterial Growth and Strain Identification

*Aeromonas hydrophila* bacteria was isolated from a deep wound infection (Strain AHB0117), a polymicrobial liver abscess on a background of cholangiocarcinoma (Strain AHB0148), a polymicrobial surgical site of infection (Strain AHB0116), diarrhea fecal samples (Strain AHB0139), and a scalp abscess due to trauma (Strain AHB0147), all de-identified. All strains were cultured in nutrient broth or agar (Oxoid, Australia) at 37°C aerobically. The bacterial strains were initially identified by Matrix Assisted Laser Desorption/Ionization – Time of Flight (MALDI-TOF; Bruker Daltonik, Germany). Conclusive identification was achieved by sequencing of the 16S rRNA region (see Table 1 for PCR conditions), as well as screening for intrinsic antibiotic resistance markers endogenous to *A. hydrophila* (see below). The 16s rRNA amplicons were purified using QIAquick^®^ PCR purification kits (Qiagen, Australia) and Sanger sequenced by the Australian Genome Research Facility (AGRF) in Queensland, Australia. The strains that were identified as *A. hydrophila* were used for subsequent bacteriophage screening.

**TABLE 1:** Primers and PCR reaction conditions for bacteria and antibiotic resistance characterization.

Oligo name	Sequence (5′ – 3′)	Cycling conditions
16S rRNA	U27F: AGAGTTTGATCMTGGCTCAG	Hold: 95°C, 3 min 32 cycles of 95°C, 30 s; 60°C, 30 s; 72°C, 90 s
	U492R: AAGGAGGTGWTCCARCC	
CphA beta-lactamase class B	FP: ACTCCATGGTCTATTTCGGG	Hold: 95°C, 10 min 35 cycles of 95°C, 30 s; 54°C, 30 s; 72°C, 45 s; 72°C, 10 min
	RP: GTCTTGATCGGCAGCTTCAT	
FOX/MOX beta lactamase class C	FP: TACTATCGCCAGTGGACGCC	Hold: 95°C, 10 min 35 cycles of 95°C, 30 s; 54°C, 30 s; 72°C, 45 s; 72°C, 10 min
	RP: TCCGCCGAGCTGGTCTTGAT	
OXA-12 beta lactamase class D	FP: TTTCTCTATGCCGACGGCAA	Hold: 95°C, 10 min 35 cycles of 95°C, 30 s; 54°C, 30 s; 72°C, 45 s; 72°C, 10 min
	RP: GTTGCCGTAGTCAAAACGGT	
Chloramphenicol resistance	FP: ATCACCTGGTTCCTGTTCAG	Hold: 95°C, 10 min 35 cycles of 95°C, 30 s; 54°C, 30 s; 72°C, 45 s; 72°C, 10 min
	RP: TACCGACGATGACCGCATAA	
MFS transporter	FP: TTCTTCGTGGTGATGCCCAT	Hold: 95°C, 10 min 35 cycles of 95°C, 30 s; 53°C, 30 s; 72°C, 45 s; 72°C, 10 min
	RP: GAAGATCAGCATCACCTGGA	
Type I-E CRISPR-associate protein Cas5/casD	FP: AACCCTACCTGCTACTATGG	Hold: 95°C, 10 min 35 cycles of 95°C, 30 s; 51°C, 30 s; 72°C, 45 s; 72°C, 10 min
	RP: ATTCTGGTGACAACGGGCAA	

### Antibiotic Sensitivity, Intrinsic Antibiotic Resistance, and CRISPR Characterization

Antibiotic sensitivity of the *A. hydrophila* clinical strains used in this study was assessed using the VITEK^®^ 2 analyzer (bioMérieux, Australia), according to the manufacturer’s instructions. Whole genome sequences of *A. hydrophila* strains published in the GenBank NCBI database were imported into CARD (Comprehensive Antibiotic Resistance Database) (Jia et al., 2017) and analyzed for genes coding antibiotic resistance. These genomes were also screened for CRISPR coded sequences using CRISPRFinder (Grissa et al., 2007). Identified sequences from (version 9.5.4) multiple strains were aligned in CLC genomic workbench and PCR primers designed from their conserved regions. The primer sequences and their PCR conditions are listed in Table 1 and amplicons were confirmed by sequencing (AGRF, Australia). Bacteriophage whole genome sequences were also screened for antimicrobial resistance genes (ARGs) and CRISPR as above.

### Bacteriophage Isolation and Host Range

Wastewater and fishpond samples from Victoria, Australia, were screened for bacteriophages by enriching the samples with *A. hydrophila*. In brief, 100 μL of log phase *A. hydrophila* was added to 10 mL of broth with 1 mL of filtered sample (0.2 μm cellulose acetate; Advantec, Australia). This enrichment was incubated for 4 days before filtration, and 10 μL of this filtrate was then spotted onto a bacterial lawn of *A. hydrophila* on agar to screen for the presence of plaques. Host range testing was performed on five clinical strains of *A. hydrophila*.

### One-Step Growth Analysis

*Aeromonas hydrophila* strains in exponential growth phase, collected by centrifugation at 12,000 × *g* for 10 min and resuspended in fresh nutrient broth at a concentration of 0.6U (OD_600_), were used for the one step growth experiments (Wang et al., 2016). The strain AHB0147 was used for one-step growth experiments involving LAh1–LAh5 bacteriophages while LAh6–LAh10 bacteriophage one-step growth experiments were performed using the strain AHB0116. One hundred microliters of bacteriophage were added to 900 μL of each *A. hydrophila* strain at a MOI of 0.01 and incubated at 4°C for 30 min to allow for adsorption. Adsorbed bacteriophage were collected by centrifugation at 12,000 × *g* for 10 min and pellet resuspended in 50 mL of fresh nutrient broth. The mixture was incubated aerobically at 37°C and bacteriophages assayed using aliquots collected every 5 min by centrifugation at 12,000 × *g* for 2 min at 4°C.

### Transmission Electron Microscopy

Bacteriophage particles were visualized by Transmission Electron Microscopy using a JEOL JEM-2100 transmission electron microscope (TEM) at 200 kV. Bacteriophage lysate was adsorbed onto 400-mesh formvar and carbon coated copper grids (ProSciTech, Australia) for 1 min. Grids were rinsed with milli-Q water and adsorbed phage particles were negatively stained twice using 2% (W/V) uranyl acetate (Sigma-Aldrich^®^, Australia) for 20 s. Excess stain was removed using filter paper and grids air dried for 30 min. Images were captured on a Gatan Orius SC200D 1 wide-angle camera using the Gatan Microscopy Suite and Digital Micrograph Imaging software (version 2.3.2.888.0). The images obtained were further analyzed using ImageJ (version 1.8.0_112).

### Bacteriophage DNA Extraction

All chemicals were purchased from Sigma-Aldrich^®^ (Australia), unless stated otherwise. Concentrated bacteriophage stock (approximately 10^11^ PFU mL^–1^) was treated with 5 mmol L^–1^ of MgCl_2_ as well as RNase A and DNase I (Promega, Australia) to a final concentration of 10 μg mL^–1^. The digest was incubated at room temperature for 30 min before polyethylene glycol precipitation at 4°C using PEG-8000 at 10% (w/v) and sodium chloride (1 gL^–1^). Precipitated virions were recovered by centrifugation at 12,000 × *g* for 5 min to obtain a pellet which was then resuspended in 50 μL nuclease free water (Promega, Australia). Viral proteins were digested with 50 μg mL^–1^ of proteinase K, 20 mmol L^–1^ EDTA and 0.5% (v/v) of sodium dodecyl sulfate for 1 h at 55°C to release phage DNA. Bacteriophage DNA was separated from proteins by addition of an equal volume of phenol-chloroform-isoamyl alcohol (29:28:1) and carefully collecting the aqueous phase after centrifugation at 12,000 × *g* for 10 min. An equal volume of isopropanol and overnight incubation at −20°C was used to precipitate bacteriophage DNA. Bacteriophage DNA was then collected by centrifugation (12,000 × *g* for 5 min) before washing in 70% ethanol, air-drying, and re-suspending in 30 μL of nuclease free water (Promega, Australia).

### Bacteriophage Whole Genome Sequencing and *in silico* Analysis

Nextera^®^ XT DNA sample preparations kits were used to prepare phage DNA for sequencing according to the manufacturer’s instructions. Whole genome sequencing of the prepared libraries was performed on an Illumina MiSeq^®^ using a MiSeq^®^ V2 300 cycle reagent kit. Sequence reads were imported into CLC genomics workbench (version 9.5.4) and assembled *de novo*. Open reading frames (ORFs) were predicted and translated using CLC genomics workbench (version 9.5.4). Translated ORFs were analyzed using BLASTP (Mount, 2007) and tRNAs and tmRNAs predicted using ARAGORN (Laslett and Canback, 2004) and tRNAscan-SE 2.0 (Lowe and Chan, 2016). Bacteriophage genomes were also analyzed for CRISPR sequences using the CRISPR database (Grissa et al., 2007). Whole genome alignments of isolated bacteriophages and those against other *Aeromonas* spp. (sourced from GenBank) were conducted and a phylogenetic tree constructed by neighbor joining method with 1000 bootstrap replicates in CLC genomics workbench (version 9.5.4). The bacteriophage genomes were also assessed by MAUVE plugin (Darling et al., 2004) in Geneious (version 11.0.5)^1^.

### Biofilm Degradation Assays

The capacity to disrupt *A. hydrophila* biofilms was determined by growing *A. hydrophila* mono-biofilms in a 96 well polystyrene plate (Greiner bio-one, Australia). The 96 well plates were inoculated with 100 μL of 10^8^ CFU mL^–1^ log phase *A. hydrophila* in broth culture and a further 100 μL of sterile broth added. The cultures were then incubated aerobically at 37°C, shaking, for 4 days. Ten microliters of bacteriophage at a concentration of 10^8^ PFU mL^–1^ was added to the established biofilms and for each experiment, heat inactivated (autoclaved) bacteriophage was used as a control to confirm that the effects on biofilm were the result of bacteriophage particles, rather than chemical residues or other matter in the preparation. Bacterial attachment was assayed according to Merritt et al. (2005). Briefly, plates were submerged in water to wash cells for 5 min before staining with 200 μL of 0.1% crystal violet for 10 min. The excess crystal violet was removed by submerging the plates in water for 5 min. The stained adherent cells were then solubilized in 70% ethanol and the absorbance in each well determined (at a wavelength of 550 nm) using a FlexStation 3 plate reader (Molecular devices, United States). Bacteriophages LAh7, LAh9, and LAh10 were tested on *A. hydrophila* strain AHB0116 biofilm whilst LAh1 was tested on biofilm formed by AHB0147.

### Viability of Biofilm

*Aeromonas hydrophila* biofilms grown on glass slides were stained with 100 μL of SYBR^®^ gold (Eugene, OR, United States; 1 mg mL^–1^) diluted in dimethyl sulfoxide (Sigma-Aldrich^®^, Australia) and 3 μL of 1 mg mL^–1^ propidium iodide (PI) in nuclease free water (Promega, Australia) for 30 min in the dark. The live/dead stained cells were mounted with 10 μL Vectorshield^®^ (Burlingame, CA, United States) on coverslips. The stained slides were visualized with an Olympus Fluoview Fv10i-confocal laser-scanning microscope (Olympus Life Science, Australia). PI stained DNA of membrane-compromised cells red while SYBR Gold^®^ stained DNA from both intact and membrane-compromised cells green.

### Statistical Analysis

Statistical tests were used to assess the capacity of bacteriophages to break down biofilms from clinical strains of *A. hydrophila*. Firstly, the Shapiro–Wilk test was used to assess whether, for each individual bacteriophage, biofilm absorbance at OD_550__nm_ was normally distributed. As these data were found to be non-normally distributed, biofilm absorbance at OD_550__nm_ for each phage was summarized in terms of the median rather than the mean, with the full five-number summaries presented in side-by-side boxplots. The interquartile range (IQR) was also calculated. Due to the non-normality of the data, the biofilm absorbance at OD_550__nm_ for each bacteriophage was compared with that for every other phage using a non-parametric test – the Wilcoxon signed-rank test. *p*-values less than 0.05 were considered to be statistically significant. All statistical tests were performed in SPSS version 24 (SPSS Inc., United States).

## Results

### Antibiotic Resistance in *Aeromonas hydrophila*

**FIGURE 1:**
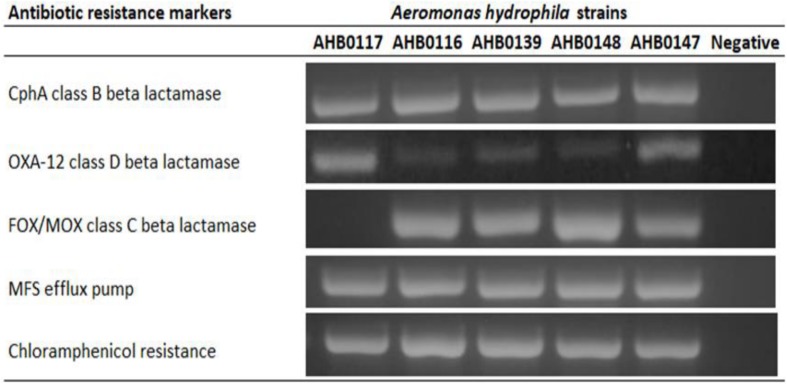
PCR detection of antibiotic resistance markers in clinical strains of *A. hydrophila* used in this study. All resistance genes were present in all clinical strains except class C beta lactamase in AHB0117. The images presented here are taken from two separate gels, which are displayed in Supplementary Figures S1,S2. The white space between these images delineates sections that were cropped from different regions of the gels in Supplementary Figures S1,S2.

Five clinical isolates identified as *A. hydrophila* were screened for ARGs by PCR amplification, revealing intrinsic multi-antibiotic resistance. These included genes coding for chloramphenicol resistance (5/5), major facilitator superfamily efflux transporter (5/5), CphA class B beta lactamase (5/5), FOX/MOX class C beta lactamase (4/5), and OXA-12 class D beta lactamases (5/5) (Figure 1). All strains used in this study were susceptible to ciprofloxacin, cotrimoxazole, and gentamicin.

### Isolation of Novel Bacteriophages Against Clinical Strains of *Aeromonas hydrophila*

**FIGURE 2:**
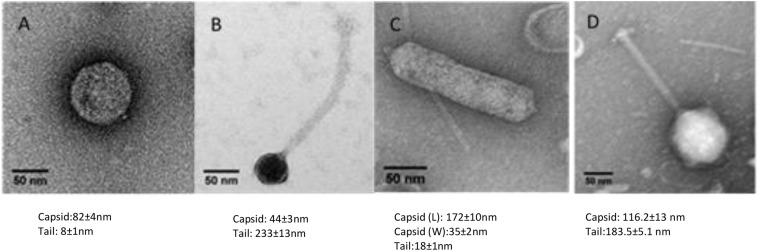
Transmission electron microscopy of representative bacteriophages from LAh1–LAh10. **(A)** Typical morphology of LAh1–LAh5 (icosahedral *Podoviridae*); **(B)** LAh7 (*Siphoviridae*); **(C)** typical morphology of LAh6, LAh8, and LAh9 (elongated *Podoviridae*); and **(D)** LAh10 (*Myoviridae*). All scale bar at 50 nm.

**FIGURE 3:**
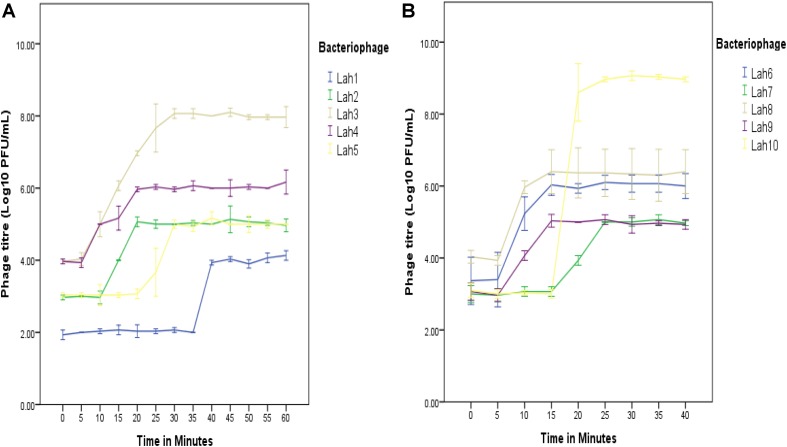
One-step growth curves for *A. hydrophila* bacteriophages LAh1–LAh10 on two separate bacterial strains (depending on host range specificity). On the same host (*A. hydrophila* AHB0147), replication kinetics were different for LAh1–LAh5 **(A)**. LAh6–LAh10 differed in their growth kinetics when grown on *A. hydrophila* strain AHB0116 **(B)**. The standard errors of the mean are calculated from three independent experiments.

Wastewater and pond samples collected from the cities of Bendigo and Melbourne, Victoria, Australia were screened for bacteriophages. Ten bacteriophages against five clinical strains of *A. hydrophila* were isolated. Of these, eight were *Podoviridae* (comprising five icosahedral and three elongated *Podoviridae*), one was a *Siphoviridae* and one was a *Myoviridae* virus. The novel bacteriophages were labeled LAh1–LAh10, with LAh1–LAh5 representing the icosahedral *Podoviridae*, LAh6, LAh8, and LAh9 representing the elongated version of the *Podoviridae*, while LAh7 and LAh10 were *Siphoviridae* and *Myoviridae* bacteriophages respectively (Figure 2). The elongated *Podoviridae* bacteriophages had capsid length ≈ 172 ± 10 nm, width ≈ 35 ± 2 nm and tail length ≈ 18 ± 1 nm while the icosahedral *Podoviridae* had capsid diameter ≈ 82 ± 4 nm and tail ≈ 8 ± 1 nm. The *Siphoviridae* bacteriophages had capsid length and tail length of ≈ 44 ± 3 nm and ≈232 ± 13 nm, respectively. *Myoviridae* bacteriophages had capsid diameter of ≈ 116 ± 13 nm and tail length ≈ 183 ± 5 nm (Figure 2). Specific features of the bacteriophages and their characteristics are summarized in Table 2 while their respective one-step growth curves are shown in Figure 3.

**TABLE 2:** LAh1–LAh10 genotypic and phenotypic characteristics.

Phage name	Source	EM morphology	Genome size (bp)	ORFs	GC%	Host range (bacterial strain)					Number of tRNAs	GenBank accession
						AHB0148	AHB0147	AHB0139	AHB0117	AHB0116		
LAh1	Wastewater	*Podoviridae*	42002	45	59.30	No	Yes	No	No	No	0	MK838107
LAh2	Wastewater	*Podoviridae*	42008	45	59.30	No	Yes	No	No	No	0	MK838108
LAh3	Wastewater	*Podoviridae*	42002	50	59.30	No	Yes	No	No	No	0	MK838109
LAh4	Wastewater	*Podoviridae*	42002	52	59.30	No	Yes	No	No	No	0	MK838110
LAh5	Wastewater	*Podoviridae*	41985	53	59.30	No	Yes	No	No	No	0	MK838111
LAh6	Fish pond	*Podoviridae*	101437	165	42.30	Yes	No	Yes	No	Yes	21	MK838112
LAh7	Wastewater	*Siphoviridae*	61426	75	61.90	No	No	Yes	Yes	Yes	0	MK838113
LAh8	Wastewater	*Podoviridae*	97408	143	42.20	Yes	No	Yes	No	Yes	21	MK838114
LAh9	Wastewater	*Podoviridae*	97988	147	42.40	Yes	No	No	No	Yes	18	MK838115
LAh10	Wastewater	*Myoviridae*	260310	227	47.50	No	No	Yes	No	Yes	4	MK838116

### Whole Genome Sequencing of Bacteriophages LAh1–LAh10

Illumina sequencing revealed novel and diverse genomes with several displaying a similar size of approximately 42,000 bp (LAh1–LAh5). While the genomes of LAh1–LAh5 were the most similar to each other, specific differences were seen, and these resulted in non-synonymous amino acid changes (differences in their genomes and amino acid sequences are highlighted in Table 3). Larger genomes were found in LAh7 (61,426 bp), LAh8 (97,408 bp), LAh9 (97,988 bp), and LAh6 (101,437 bp). LAh10 had the largest genome (260,310 bp) which is the also the largest reported to date against *A. hydrophila*. The genomes for LAh1–LAh10 were annotated and submitted to GenBank with accession numbers shown in Table 2. All bacteriophages had their genomes in a linear topology except LAh6 which had a circularly permuted genome. The number of ORFs varied with the genome size and ranged from 45 to 227 (Table 2). The GC% content ranged from 42.2% in LAh8 to 61.9% in LAh7. While no bacteriophage showed presence of tmRNA in their genomes, tRNAs were present in 4/10 of the bacteriophages (Table 2). The number or type of tRNAs were not associated with genome size. LAh6 and LAh8 had the highest number of tRNAs at 21 each, followed by LAh9 with 18 tRNAs and LAh10 with 4 tRNAs. Therefore, the bacteriophages with a lower GC% content had a higher number of tRNAs (Table 2). Bacteriophages LAh1–LAh5, and LAh7 did not have any tRNAs in their genomes. None of the bacteriophages isolated in this study had genes coding for putative CRISPR sequences, ARGs, toxins or chromosome integration genes in their genomes.

**TABLE 3:** Differences in the genomes of bacteriophages LAh2–LAh5 compared to LAh1.

Bacteriophage	Nucleotide change	Position	Non-synonymous amino acid change	Putative protein
LAh2; LAh4	C > A	1939	Z > K	Scaffolding protein
LAh5	A > G	8203	R > G	Hypothetical
LAh5	G > C	8205	R > G	Hypothetical
LAh5	Deletion (18 bp)	8207..8224	R; P; S; R; TGA (STOP); S	Hypothetical
LAh4	G > A	14009	A > T	Tail fiber
LAh2; LAh4	A > G	15068	N > D	Tail fiber
LAh3; LAh5	A > G	15075	C > Y	Tail fiber
LAh2	Insertion 4 bp	33001..33006	I and M (START)	DNA polymerase

### Bacteriophage Phylogeny and Host Specificity

**FIGURE 4:**
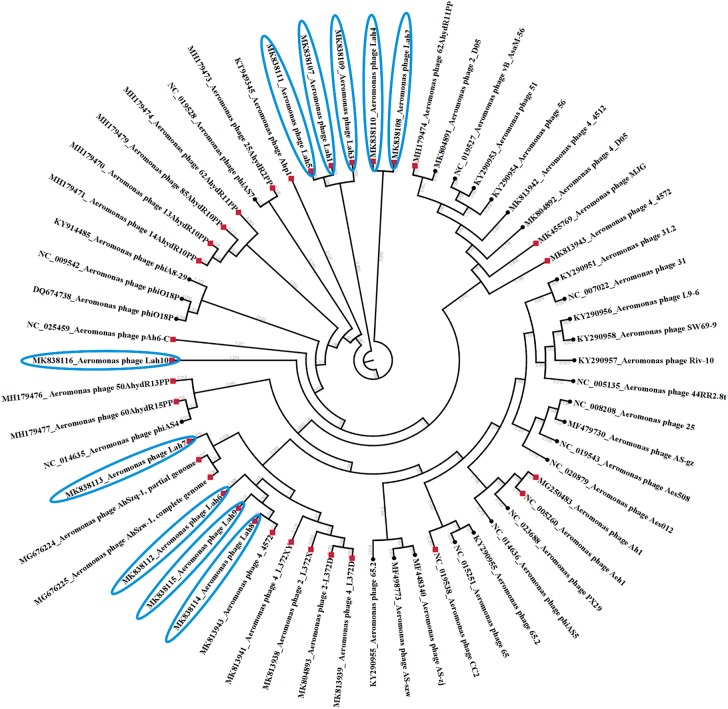
Phylogenetic tree showing genetic relatedness of bacteriophages against *Aeromonas* species. All bacteriophages against *A. hydrophila* are indicated by red diamond nodes including those isolated in this study using clinical strains of *A. hydrophila* which have been highlighted in blue.

Prior to this study there were 30 reported bacteriophages against *Aeromonas* spp. including eight against *A. hydrophila* which have had their genome completely sequenced. The 10 bacteriophages reported here are the first lytic bacteriophages to be isolated against clinical strains of *A. hydrophila*. The complete genome sequences of the previously isolated bacteriophages against *Aeromonas* spp. sourced from GenBank and those from this study were compared. Figure 4 reveals the clustering of bacteriophages against clinical strains of *A. hydrophila* forming two clusters among other bacteriophages isolated using *A. hydrophila* strains from the environment but further away from those against other *Aeromonas* species.

**FIGURE 5:**
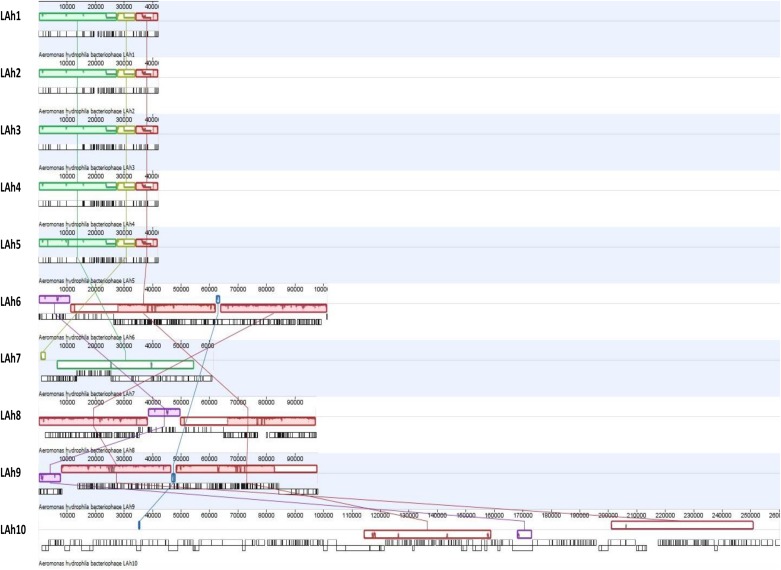
Whole genome alignment of LAh1–LAh10 from top to bottom showing similarities and differences between genomes. Each colored colinear block represents a conserved region across the genomes, and these are connected by colored matching lines to trace homology between genomes. Colinear blocks that are offset represent regions that are expressed in the opposite orientation. The uncolored gaps between and within the colinear blocks represent differences between genomes or differences within conserved regions across genomes. The origin of individual ORFs is represented by vertical black lines below the colinear blocks (detailed annotation and putative functionality of each ORF can be accessed through GenBank with accession numbers provided in Table 2). LAh1–LAh5 share all their conserved regions (represented by green, yellow, and red blocks) with differences indicated by nicks and gaps within those blocks. Two of the three colinear blocks from LAh1–LAh5 are shared with LAh7 (green and yellow) and one (red) with all the other bacteriophage genomes (LAh6, LAh8, LAh9, and LAh10). LAh6, LAh8, and LAh9 share homology in three blocks (red, pink, and purple), while LAh6 and LAh9 share a fourth region (blue) which is also found in LAh10 but not LAh8. The purple block is conserved between LAh6, LAh8, LAh9, and LAh10.

These differences among the isolated bacteriophage genomes were further analyzed by Mauve whole genome alignment of LAh1–LAh10 (Figure 5). The differences in the bacteriophage cluster of LAh1–LAh5 is detailed in Table 3 to highlight functional differences between these bacteriophages. For the cluster of bacteriophages LAh6, LAh8, and LAh9, the Mauve alignment showed the presence of a genetic segment (Blue colored colinear block in Figure 5) in the region of the putative tail fiber genes of bacteriophages that possibly contributed to their differences in host range. While similar in their genomes, LAh6 and LAh8 are able to lyse *A. hydrophila* strain AHB0139 that LAh9 is not (Table 2). The genome alignments show close homology between LAh1 and LAh5, and a diversity of nucleotide sequence between genomes of LAh7 and LAh10.

### Capacity of LAh1–LAh10 to Disrupt *Aeromonas hydrophila* Biofilms *in vitro*

**FIGURE 6:**
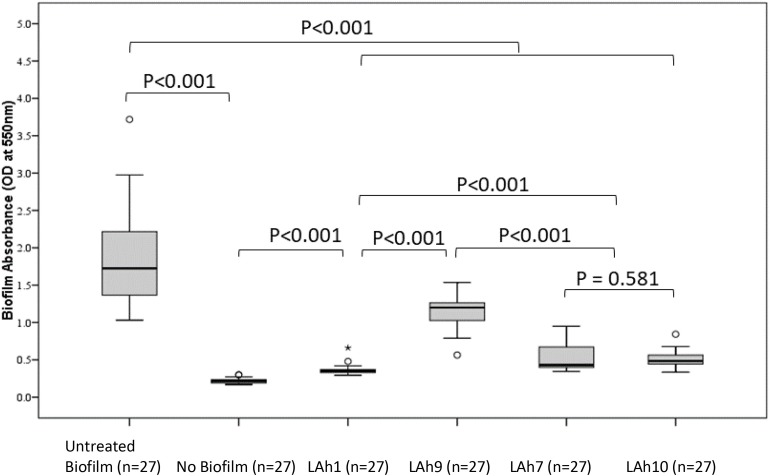
*Aeromonas hydrophila* biofilm absorbance measurements after treatment with bacteriophages LAh1, LAh7, LAh9, and LAh10. Bacteriophages LAh7, LAh9, and LAh10 were tested on *A. hydrophila* strain AHB0116 biofilm whilst LAh1 was tested on biofilm formed by AHB0147. Bacteriophages with higher GC% content and lower numbers of tRNAs [LAh1 (59.30%; 0), LAh7 (61.90%; 0), and LAh10 (47.50%; 4)] showed significantly greater capacity to degrade biofilms (*p* < 0.001) than that with lower GC% content and higher numbers of tRNAs [LAh9 (42.40%; 18)]. * Is an extreme outlier.

The capacity to disrupt *A. hydrophila* biofilms was analyzed quantitatively by evaluating the biofilm mass remaining after bacteriophage treatment and estimating the viability of the remnant biofilm. A representative bacteriophage from each morphological group was analyzed for capacity to disrupt biofilm. LAh1 was used as an example of icosahedral *Podoviridae*, LAh9 for the elongated *Podoviridae*, LAh7, a *Siphoviridae*, and LAh10 a *Myoviridae*. All biofilm experiments were performed using *A. hydrophila* bacterial strain AHB0116 that was lysed by all bacteriophages isolated here except for LAh1, in which case the host AHB0147 was used. In all cases, the biofilm mass was significantly reduced in bacteriophage treated compared to non-treated groups (*p* < 0.001). The untreated biofilm had a median (IQR) absorbance at OD_550__nm_ of 1.73 (0.86) whilst the largest value for the remnant biofilm after bacteriophage treatment was 1.20 (0.27), *p* < 0.001 (following treatment with LAh9). Treatment with LAh1 resulted in the lowest absorbance for the remnant biofilm [median (IQR) at OD_550__nm_ of 0.35 (0.04)], significantly lower (*p* < 0.001) than those treated with all the other bacteriophages. LAh7 and LAh10 had statistically similar (*p* = 0.58) remaining biofilm with absorbance of median (IQR) at OD_550__nm_ of 0.43 (0.29) and 0.48 (0.13), respectively (Figure 6). Of the bacteriophages tested on the *A. hydrophila* biofilms, those with higher GC% content and lower numbers of tRNAs [LAh1 (59.30%; 0), LAh7 (61.90%; 0), and LAh10 (47.50%; 4)] showed significantly greater capacity to degrade biofilms (*p* < 0.001) than that with lower GC% content and higher numbers of tRNAs [LAh9 (42.40%; 18)] (Figure 6).

### Viability of Biofilm Treated With Bacteriophages

**FIGURE 7:**
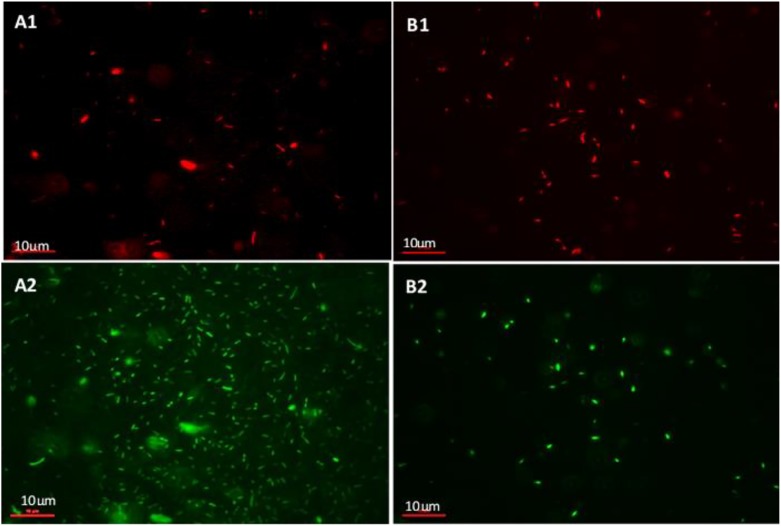
Live/dead staining of *A. hydrophila* biofilm using propidium Iodide (PI) and SYBR gold^®^. **(A1,A2)** show untreated *A. hydrophila* biofilm on a glass slide. **(A1)** PI stained (dead) cells and **(A2)** cells stained with SYBR gold^®^ (total population of cells). **(B1,B2)** *A. hydrophila* biofilm on a glass slide treated with bacteriophage LAh1 for 60 min. **(B1)** PI stained (dead) cells and **(B2)** cells stained with SYBR gold^®^ (total population of cells).

The viability of the biofilm mass was investigated using SYBR Gold^®^ and PI live/dead staining. Figure 7 shows cells fluorescing green (whole population of cells making up the biofilm) and red (dead, membrane-compromised cells). The icosahedral *Podoviridae* Bacteriophage LAh1 on host strain AHB0147 was used in the biofilm viability experiments. Figure 7 shows untreated biofilm with a sparse population of membrane-compromised cells (Figure 7B1) compared to a dense total population (Figure 7B2). This indicated that cell population in the untreated biofilm was comprised of mostly membrane-intact cells. This was in contrast to the biofilms treated with bacteriophage (Figures 7A1,A2) in which the density was comparable between total population and membrane-compromised cells implying the cell population was mostly dead. In the bacteriophage treated biofilm, the total population and dead cells were both sparse. Treatment with heat inactivated (autoclaved) bacteriophage did not affect biofilm growth, indicating that *Aeromonas* biofilm disruption was the result of bacteriophage particles, and not other material in the preparation.

## Discussion

Increasing global temperatures have allowed a number of micro-organisms to emerge as potential causes of disease (Aguirre and Tabor, 2008). *A. hydrophila* is one bacterial species benefiting from global warming with more clinical strains emerging (Azzopardi et al., 2011). These bacteria are usually resistant to first and second line antibiotic therapy involving beta-lactam drugs and third generation cephalosporins via mechanisms such as resistance genes and biofilm formation (Janda and Abbott, 2010). Bacteriophages, which have been suggested as an alternative to antibiotics, have been isolated against environmental and fish pathogen strains of *A. hydrophila* (Chow and Rouf, 1983; Merino et al., 1990a, b; Gibb and Edgell, 2007; Shen et al., 2012; Jun et al., 2013; Anand et al., 2016; Wang et al., 2016; Le et al., 2018; Yuan et al., 2018; Bai et al., 2019; Cao et al., 2019; Kazimierczak et al., 2019). The host range of these bacteriophages, however, was not reported to extend to clinical strains. The current study is the first to report the isolation and characterization of bacteriophages lytic against clinical strains of *A. hydrophila*, all of which carry intrinsic antibiotic resistance markers. These bacteriophages (LAh1–LAh10) displayed diversity in their morphology and genomic composition. The genomes of LAh1–LAh5 were the most similar to each other, yet specific differences were seen, and these resulted in non-synonymous amino acid changes. These changes may have contributed to the differences in growth kinetics observed between LAh1 and LAh5. Phylogenetic comparison between LAh1 and LAh10 and other *Aeromonas* bacteriophages revealed that LAh1–LAh10 clustered separately to those lytic for environmental strains of *A. hydrophila* and to bacteriophages against other species of *Aeromonas*.

*Aeromonas hydrophila* has been shown to thrive in water storage tanks and swimming pools as biofilms (Julia Manresa et al., 2009; Chubaka et al., 2018). The persistence of *A. hydrophila* in such environments has been implicated in several diseases such as diarrhea, sepsis and necrotizing fasciitis (Janda and Abbott, 2010). Bacteria growing in biofilm communities are more difficult to control than planktonic ones and *A. hydrophila* in particular is resistant to treatment with antibiotics and disinfectants such as chlorine (Donlan and Costerton, 2002; Janda and Abbott, 2010). Bacteriophages, which have been shown to be safe in clinical applications (Malik et al., 2017; Dedrick et al., 2019; Nir-Paz et al., 2019; Schmidt, 2019) may provide a useful solution to controlling this bacterium in a changing climate. The bacteriophages isolated and tested in this study were able to significantly reduce the *A. hydrophila* biofilm mass after 24 h of treatment. While these bacteriophages as well as others are active against biofilms (Hansen et al., 2019; Saha and Mukherjee, 2019), the factors associated with their efficacy have not been fully elucidated. The bacteriophages isolated in this study were diverse in morphology, genome content and organization. Their *Podoviridae*, *Siphoviridae*, or *Myoviridae* morphology was not associated with their capacity to disrupt a biofilm. The size of the genome and physical size of the bacteriophages was also not associated with their biofilm disruptive capabilities. However, bacteriophages in this study with a low GC% content and with a higher number of tRNAs had a lower biofilm disruptive efficacy. While we did not assess the GC% content of the *A. hydrophila* strains used for the biofilm assays here, the GC% of *A. hydrophila* published genomes ranged from 60.2 to 61.3% (Chan et al., 2015; Tan et al., 2015a, b; Forn-Cuni et al., 2016a, b; Moura et al., 2017). Assuming our bacterial strains had similar content, then it would appear that bacteriophages in our study which matched more closely the GC% of the bacteria were significantly more successful in degrading biofilms. Bacteria growing in biofilms will slow down their metabolism (Donlan and Costerton, 2002), and this factor may have a greater impact on those bacteriophages that carry their own tRNAs. It is important, however, to highlight that the sample of bacteriophages we assayed in our study was small, and the number of those with and without tRNAs was even smaller. Therefore, more concrete experimental data and extensive sampling is required before definitive conclusions can be drawn from such observations.

While others report that bacteriophage GC% content was related to GC% content of host bacteria (Xia and Yuen, 2005) and that presence of tRNAs was characteristic of more virulent bacteriophages (Bailly-Bechet et al., 2007), in our study there was no apparent relationship between host range and GC% content or number of tRNAs present in bacteriophage genomes. In the bacteriophages isolated here, those with lower GC% content had higher numbers of tRNAs, similar to findings in a study of bacteriophages against *Aeromonas salmonicida* subsp. *Salmonicida* (Vincent et al., 2017). This may not be surprising if we assume that the genomes of *A. hydrophila* used here have a similar GC% content to those previously published [60.2–61.3% (Chan et al., 2015; Tan et al., 2015a, b; Forn-Cuni et al., 2016a, b; Moura et al., 2017)] and that while bacteriophage genomes evolve to match the GC% of their hosts (Xia and Yuen, 2005), those whose GC content is lower may require tRNAs to complement their biochemical requirements. Finally, certain bacteriophages may code for and transfer ARGs in bacteria through transduction (Gunathilaka et al., 2017; Brown-Jaque et al., 2018; Larranaga et al., 2018; Wang et al., 2018). None of the bacteriophages isolated here were found to code for these, which may be a favorable feature if they were to be used in environmental or clinical settings.

## Conclusion

We report here a diverse range of novel bacteriophages, LAh1–LAh10, which are the first shown to be active against clinical strains of *A. hydrophila*. While these bacteria may survive decontaminating efforts in water by quorum sensing and forming biofilms, bacteriophages offer the potential of an alternative to control their growth in the environment, as well as following human infection. Functionally, the bacteriophages tested here were capable of *A. hydrophila* biofilm disruption.

## Data Availability Statement

The complete genome sequences have been submitted to NCBI GenBank with accession numbers: MK838107, MK838108, MK838109, MK838110, MK838111, MK838112, MK838113, MK838114, MK838115, and MK838116.

## Author Contributions

JT, HC, and MK: conceptualization. MK, HK, and HC: data collection. MK and TB: genomics. MK, LS, and PL: electron microscope imaging and biofilm. MK, ML, and JT: data analysis. JT, HC, and SP: supervision. MK and JT: writing – original draft. MK, TB, HC, and JT: writing – review and editing.

## Conflict of Interest

The authors declare that the research was conducted in the absence of any commercial or financial relationships that could be construed as a potential conflict of interest.

## Footnotes

1 http://www.geneious.com/
