# Supplementary material for: Novel Bacteriophages Capable of Disrupting Biofilms From Clinical Strains of Aeromonas hydrophila
Source: Front Microbiol. 2020 Feb 14;11:194. doi: 10.3389/fmicb.2020.00194 (PMC7033617; doi:10.3389/fmicb.2020.00194)
Supplement: Supplementary file 1 [file Image_1.pdf]

Title: **Novel bacteriophages capable of disrupting biofilms from clinical strains of *Aeromonas hydrophila*.**

Mwila Kabwe[1], Teagan Brown[1], Lachlan Speirs[1], Heng Ku[1], Michael Leach[2] Hiu Tat Chan[3], Steve Petrovski[4], Peter Lock[5], Joseph Tucci[1*]

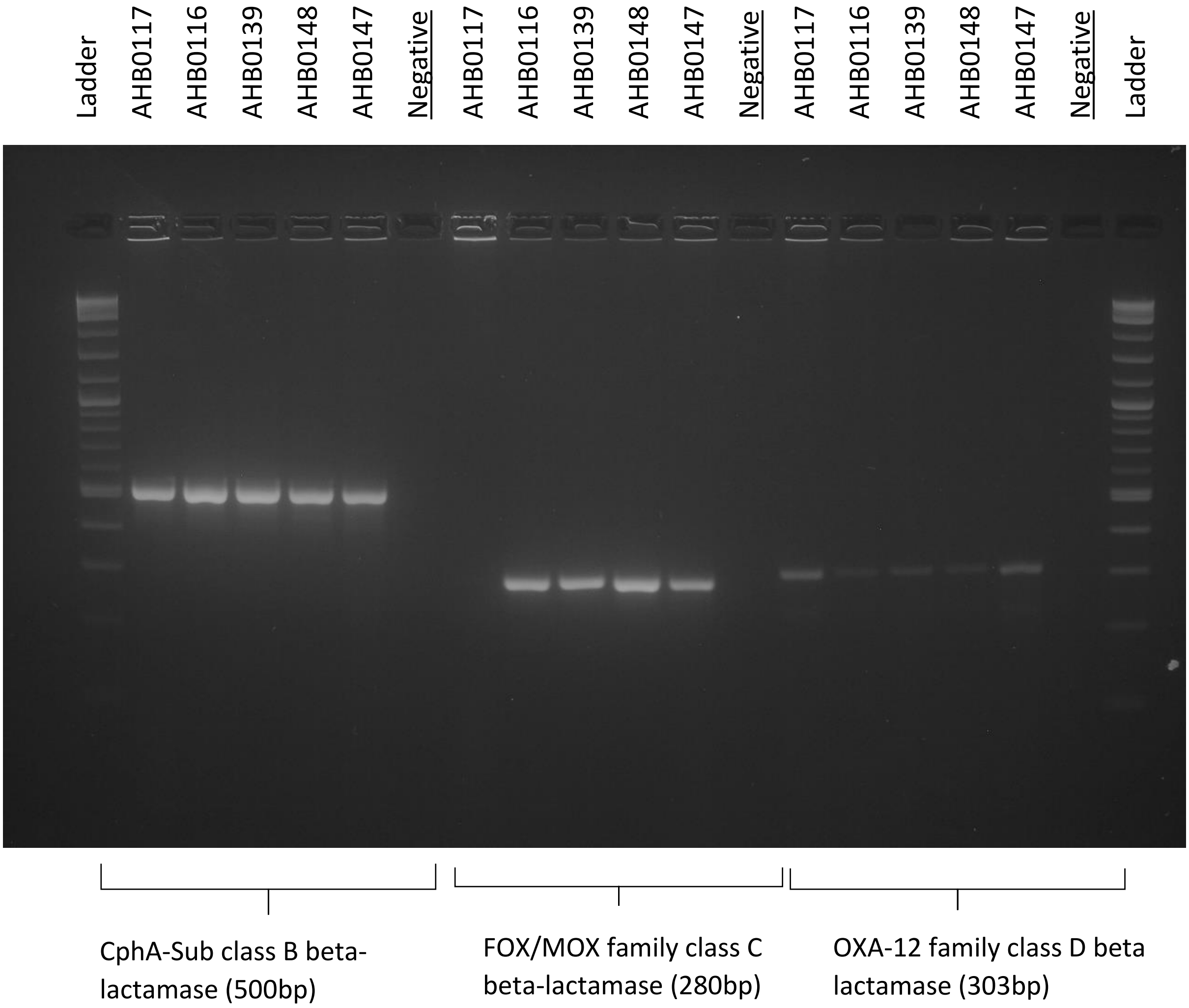

Figure S1: PCR detection of antibiotic resistance markers in clinical strains of *A. hydrophila* used in this study. CphA-sub class B beta-lactamase and OXA-12 family class D beta lactamases were detected in all strains. AHB0117 was the only strain in which the FOX/MOX family class C beta-lactamase was not detected.
